# An mRNA-Based Multiple Antigenic Gene Expression System Delivered by Engineered *Salmonella* for Severe Fever with Thrombocytopenia Syndrome and Assessment of Its Immunogenicity and Protection Using a Human DC-SIGN-Transduced Mouse Model

**DOI:** 10.3390/pharmaceutics15051339

**Published:** 2023-04-26

**Authors:** Ji-Young Park, Chamith Hewawaduge, Chandran Sivasankar, Khristine Kaith S. Lloren, Byungkwan Oh, Mi Young So, John Hwa Lee

**Affiliations:** 1 Department of Veterinary Public Health, College of Veterinary Medicine, Jeonbuk National University, Iksan 54596, Republic of Korea; 2 Department of Veterinary Pathology, College of Veterinary Medicine, Jeonbuk National University, Iksan 54596, Republic of Korea

**Keywords:** Salmonella delivery, vaccine, SFTS, viral protein, hDC-SIGN

## Abstract

Currently, there are no commercial vaccines or therapeutics against severe fever with thrombocytopenia syndrome (SFTS) virus. This study explored an engineered Salmonella as a vaccine carrier to deliver a eukaryotic self-mRNA replicating vector, pJHL204. This vector expresses multiple SFTS virus antigenic genes for the nucleocapsid protein (NP), glycoprotein precursor (Gn/Gc), and nonstructural protein (NS) to induce host immune responses. The engineered constructs were designed and validated through 3D structure modeling. Western blot and qRT-PCR analyses of transformed HEK293T cells confirmed the delivery and expression of the vaccine antigens. Significantly, mice immunized with these constructs demonstrated a cell-mediated and humoral response as balanced Th1/Th2 immunity. The JOL2424 and JOL2425 delivering NP and Gn/Gc generated strong immunoglobulin IgG and IgM antibodies and high neutralizing titers. To further examine the immunogenicity and protection, we utilized a human DC-SIGN receptor transduced mouse model for SFTS virus infection by an adeno-associated viral vector system. Among the SFTSV antigen constructs, the construct with full-length NP and Gn/Gc and the construct with NP and selected Gn/Gc epitopes induced robust cellular and humoral immune responses. These were followed by adequate protection based on viral titer reduction and reduced histopathological lesions in the spleen and liver. In conclusion, these data indicate that recombinant attenuated Salmonella JOL2424 and JOL2425 delivering NP and Gn/Gc antigens of SFTSV are promising vaccine candidates that induce strong humoral and cellular immune responses and protection against SFTSV. Moreover, the data proved that the hDC-SIGN transduced mice as a worthy tool for immunogenicity study for SFTSV.

## 1. Introduction

Severe fever with thrombocytopenia syndrome (SFTS) is a tick-borne emerging infectious disease with a 10~30% fatality rate [1]. The SFTS virus (SFTSV), the etiological agent, is directly transmitted to humans through tick bites. However, new epidemiological research data suggest that domestic animals serve as an intermediate host for SFTSV transmission to humans [2,3,4]. Since the first human case was reported in China, SFTS has emerged in neighboring countries, including Japan [5] and South Korea [6]. Several research groups have attempted to develop vaccines and antiviral therapeutics against SFTS [7,8]; however, there are no licensed vaccines against SFTS. The viral genome of SFTSV comprises three negative-stranded RNA segments, large (L), medium (M), and small (S) [9]. The L segment encodes the RNA-dependent RNA polymerase (RdRp); the M segment encodes the envelope Gn/Gc glycoproteins; and the S segment encodes two proteins, the nonstructural protein (NS) and the nucleocapsid protein (NP) [10]. Two Gn/Gc antigenic components are considered the primary targets for vaccine development due to the abundance of neutralizing epitopes [11]. In addition, the NP and NS are conserved among all *Phleboviruses* and play essential roles in cell-mediated immune responses [12,13].

An ideal vaccine vector must be engineered to deliver target antigens to stimulate the host immune response. The use of bacterial vaccine vectors for antigen delivery has been studied over the past three decades [14]. Live attenuated *Salmonella enterica* has substantial potential to deliver homologous and heterologous vaccine antigens and stimulate host mucosal, humoral, and cellular immune responses [15,16]. Attenuating live bacterial vectors can be produced by genetic manipulation of genes involved in bacterial virulence. The *lon* gene is a negative regulator of *Salmonella* pathogenic island 1 (SPI-1) that comprises multiple virulence factors and determines pathogenesis. Deletion of *lon* enables *Salmonella* to efficiently invade epithelial cells and reach lymphatic organs to induce immune boosting [17]. Additionally, disruption of the *lon* gene resulted in increased adhesion and invasion of *Salmonella* with reduced intracellular survival, which is essential for a safe and effective bacterial vaccine vector. The *cpxR* gene of the cpxA/cpxR two-component system is involved in intra-cellular survival, stress response [18,19], and biofilm formation of *Salmonella* spp. Deleting the *cpxR* gene promoted immune recognition by the host cells, resulting in enhanced immune response [20,21,22]. The aspartate semialdehyde dehydrogenase (*asd*) gene is used as an auxotrophic selective marker for the stringent retention of antigen-expressing plasmid [23]. The *sifA* gene of *Salmonella* pathogenic island 2 (SPI-2) is involved in maintaining the integrity of the *Salmonella*-containing vacuoles (SCVs). The absence of the *sifA* gene prevents the formation of SCVs, hence, facilitating the release of *Salmonella* into the host cytosol and thereby increasing major histocompatibility complex (MHC) class I presentation [24,25,26]. In a previous study, we devised a *Salmonella* mutant strain, JOL2500 (Δ*lon*, Δ*cpxR*, Δ*sifA*, Δ*asd*), and demonstrated its ability to deliver a plasmid and express antigens in vitro and in vivo [27]. In addition, we constructed a vector system that exploits the activity of the RdRp complex (nonstructural proteins 1–4) and 26 subgenomic promoters (26SP) of Semliki Forest virus (SFV) to improve the antigen expression by enhanced cytoplasmic mRNA transcription [27,28,29]. 

In the present study, we used a novel humanized immunocompetent mice model based on our recent report. In the previous study, we developed a mouse model susceptible to SFTSV infection by overexpressing the human C-type lectin receptor (hDC-SIGN) via an adeno-associated virus (AAV)-mediated transduction [30]. To assess vaccine and antiviral therapeutic efficacy, a variety of animal models have been developed, even with lethal results, but most are immunosuppressed [8,31,32,33]. Therefore, we developed a mouse model with normal immune status and susceptibility to SFTSV. We delivered human dendritic cell-specific ICAM-3-grabbing non-integrin (hDC-SIGN) as a receptor [34,35] for susceptibility to SFTSV via recombinant adeno-associated virus (AAV2) [36]. AAV2-transfected C57BL/6 mice showed stable hDC-SIGN expression in organs, and when treated with high concentrations of SFTSV, rAAVhDC-SIGN-transfected mice showed reduced platelet and leukocyte counts depending on higher viral titer and pathological signs than control mice. Therefore, we chose the humanized immunocompetent mice as an ideal model to study the immune response and protection against SFTSV. 

In the present study, we used live-attenuated *Salmonella* to deliver self-replicating mRNA vaccine constructs harboring SFTSV immunogen NP, Gn/Gc, and NS. Following immunization, we evaluated the antigen-specific humoral and cell-mediated immune responses in vivo. Using this AAV2/hDC-SIGN-transduced mouse model, we evaluated the protective efficacy of the vaccine constructs following an SFTSV challenge. 

## 2. Materials and Methods

### 2.1. Animals and Ethics Statement 

All animal experiments were approved by the Jeonbuk National University Institutional Animal Care and Use Committee (IACUC, NON2022-024-001) under the Korean Council on Animal Care and Korean Animal Protection Law guidelines 2007; Article 13 (Experiments with animals). Specific pathogen-free (SPF) C57BL/6 mice were purchased from Samtako (Osan, Gyeonggi-do, Republic of Korea) and maintained under a 12 h light–dark cycle with ad libitum access to food and water. Virus experiments were performed in a biosafety level (BSL)-3 animal facility according to guidelines of the Jeonbuk National University Institutional Biosafety Committee (JBNU2019-01-001-001).

### 2.2. Bacterial Strains, Plasmids, Primers 

All bacterial strains, plasmids, and primers used in this study are listed in Table 1. The bacteria were routinely propagated in Luria Bertani (BD Difco, Franklin Lakes, NJ, USA) medium at 37 °C with vigorous shaking using appropriate supplements and antibiotics, including diallyl phthalate (DAB) for *E. coli* 232, kanamycin for *E. coli* BL21(DE3), and ampicillin for *E. coli* DH5α.

### 2.3. Cell Lines and Virus

HEK293T (ATCC^®^ CRL3216™) and VeroE6 (ATCC CCL-81) cells were cultured in Dulbecco’s modified Eagle’s medium (DMEM, Gibco, MA, Waltham, USA), supplemented with 10% fetal bovine serum (FBS, Gibco) and 1% antibiotic-antimycotic (Gibco) at 37 °C in a 5% CO_2_ atmosphere. SFTS virus strain KADGH (NCCP43261) was purchased from National Culture Collection for Pathogens (NCCP, Cheongju, Chungbuk, Republic of Korea).

Vero cells plated into 75 cm^2^ tissue culture flasks were infected with the SFTSV strain. The cells were incubated for five days at 37 °C, followed by freezing and thawing three times. The virus was harvested and titrated in 96-well microplates using 10-fold serial dilutions. After fixing and staining the cells with specific NP hyperimmune serum against SFTSV, cells were incubated with alexafluor-488 anti-rabbit IgG (Invitrogen, Waltham, MA, USA) secondary antibody. The viral titrations were performed according to the Reed and Muench method [37] and determined as the 50% fluorescent assay infectious dose (FAID_50_)/mL [38].

### 2.4. Bioinformatic Analysis of Target Proteins

Multiple nucleotide sequences of small (S) and medium (M) segments from SFTSV were procured from the NCBI, and consensus sequences were generated using the EMBL-EBI ClustalW server. Immunogenic linear B-cell epitopes of the Gn/Gc glycoprotein were screened using the Immune Epitope Database (IEDB) analysis tool (Supplemental Figure S1). The tertiary structure of the selected antigens predicted by the ProSA server was used to validate the 3D structure and z-score [39]. Furthermore, the PROCHECK web server was implemented to produce Ramachandran plots [40,41].

### 2.5. Construction of the Eukaryotic Plasmid Expressing the Recombinant SFTSV Antigen 

Construction of the SFV replicon-based pJHL204 eukaryotic plasmid was described in a previous study [27]. Genes encoding NP, NS, Gn/Gc, and Gn/Gc epitopes were synthesized (Integrated DNA Technology, Coralville, IA, USA) and cloned into the pJHL204 vector. For preparation of recombinant proteins, each antigen was cloned into a pET28a (+) vector and purified from *E. coli* BL21 (DE3) using Ni-NTA column chromatography (TAKARA, Tokyo, Japan). The expression of purified protein fractions was examined by sodium dodecyl sulfate-polyacrylamide gel electrophoresis (SDS-PAGE). Polyclonal antibodies against each antigen were produced in SPF New Zealand white rabbits [42].

### 2.6. Immunofluorescence Assay 

Transfection with pJHL203, pJHL204, and pJHL205 expressing NP-GnGc-epitope was carried out as described in Table 1. After 48 h post-transfection, cells were fixed with 80% cold acetone (Daejung, Siheung, Gyeonggi-do, Republic of Korea) at −20 °C for 10 min. After blocking with 1% BSA for 1 h at room temperature, cells were incubated with SFTSV NP-Gn/Gc-epitope hyperimmune serum (1:1000) at 4 °C overnight. Finally, cells were incubated with alexafluor-488 anti-rabbit IgG (Invitrogen) secondary antibody; 4′,6-diamidino-2-phenylindole (DAPI, Sigma-Aldrich, St. Louis, MO, USA) was used to stain the nuclei. After three washes with PBS, the cells were examined using the Leica Fluorescence Microscope (Leica Biosystems, Wetzlar, Germany). 

### 2.7. Expression of Recombinant Antigens In Vitro

HEK293T cells were seeded into 6-well plates (1 × 10^6^ cells/well) and were transfected with pJHL204-empty as a vector control, pJHL204-NP-Gn/Gc, pJHL204-NP-P2A-Gn/Gc, or pJHL204-NS plasmids (1ug/uL) using Lipofectamine™ 3000 Transfection Reagent (Invitrogen, Waltham, MA, USA). After 48 h post-transfection, cells were lysed by RIPA buffer containing a protease inhibitor cocktail (Thermo Fisher Scientific, Waltham, MA, USA). Cell lysates were subjected to 12% SDS gel electrophoresis and transferred to a PVDF membrane (Millipore, Burlington, MA, USA). Membranes were blocked with 5% skim milk for 1 h and incubated with rabbit hyperimmune sera against NP-Gn/Gc-epitope, NP, Gn/Gc, and NS (1:1000) overnight at 4 °C, respectively. Membranes were then incubated with goat anti-rabbit HRP-IgG (Southern Biotech, Birmingham, AL, USA) or goat anti-mouse HRP-IgG (Southern Biotech) antibody (1:5000) for 1 h at room temperature. Finally, membranes were developed with Amersham ECL detection reagents (Cytiva, Marlborough, MA, USA) and visualized using an Amersham ImageQuant 800 imaging system (Cytiva). 

### 2.8. Animal Experiments 

To evaluate the immunogenicity of the vaccine constructs, five-week-old female C57BL/6 mice (*n* = 40) were randomly divided into five groups (*n* = 8), and mice in three experimental groups were immunized with PBS, JOL2424, JOL2425, and JOL2426, respectively. Mice immunized with JO2420 served as the vector control group. Mice group that received only PBS was considered as control. Mice in all groups received 1 × 10^7^ CFU/100 µL via the intramuscular (i.m.) route (Table 2). On the 2-week post-primary vaccination, mice in all groups were given a booster immunization. Blood samples (*n* = 4) were collected from the retro-orbital sinus at weeks 0, 1, 2, 4, 6, and 8 post-primary immunization and isolated the serum by centrifugation. At 2-weeks post-booster immunization, four mice from each group were sacrificed to collect splenocytes and were processed according to a previously described procedure [43]. 

To evaluate the protective efficacy, five-week-old female C57BL/6 mice (*n* = 40) were randomly divided into 4 groups and immunized with constructs as described in Supplementary Table S1. One-week post-booster inoculation mice from each group were transduced intravenously (i.v.) with rAAV-DC-SIGN particles (1 × 10^11^ vp/100 µL) [30]. At 2 weeks after the final vaccination, transduced mice (*n* = 8) were intraperitoneally (i.p.) challenged with 1 × 10^3^ FAID_50_ of SFTSV and monitored for four days. Body weight and rectal temperature were measured daily. On the 2 and 4 days post-challenge, four mice were sacrificed, and serum, spleen, and liver samples were collected to determine viral load. Serum samples were collected (*n* = 4) at days 0 and 4 post-challenge and stored at −80 °C. To analyze the platelet count, complete blood count was performed with an automatic blood cell counter (Exigo-Vet., Boule Medical AB Inc., Stockholm, Sweden) using collected blood samples.

### 2.9. Quantitative Real-Time PCR (qRT-PCR) 

The splenocytes were seeded in 24-well plates (1 × 10^6^ cells/well) and stimulated with purified NP-Gn/Gc-epitope, NP, Gn/Gc, and NS recombinant proteins (500 ng/well) for 48 h, respectively. Following stimulation, total RNA was extracted from splenocytes using the GeneAll Hybrid-R kit (GeneAll, Seoul, Republic of Korea). The cDNA was synthesized using a reverse transcription master premix kit (ELPIS-BIOTECH, Daejeon, Republic of Korea). qRT-PCR performed using EzAmp™ qPCR 2X master mix (ELPIS-BIOTECH). The relative cytokine amounts of IFN-γ, TNF-α, IL-4, IL-6, and IL-10 [44] mRNA levels with β-actin were determined by the 2^−ΔΔCT^ method [45].

**Table 1:** Plasmids, bacterial strains, and primers used in this study.

Strain/Plasmid	Description	Reference
**Plasmid**		
pJHL204	asd^+^, CMV promoter, RdRp complex, SV40 promoter, 26Spromoter, pBR322 ori	Lab stock
pJHL204-NP-Gn/Gc	asd^+^, CMV promoter, RdRp complex, SV40 promoter, 26Spromoter, pBR322 ori, NP-Gn/Gc-epitope	This study
pJHL204-NP-P2A-GnGc	asd^+^, CMV promoter, RdRp complex, SV40 promoter, 26Spromoter, pBR322 ori, NP-P2A-Gn/Gc	This study
pJHL204-NSs	asd^+^, CMV promoter, RdRp complex, SV40 promoter, 26Spromoter, pBR322 ori, NS	This study
pJHL203-NP-GnGc	asd^+^, CMV promoter, RdRp complex, ΔSV40, promoter, Δ26Spromoter, pBR322 ori, NP-Gn/Gc-epitope	This study
pJHL205-NP-GnGc	asd^+^, CMV promoter, RdRp complex, SV40 promoter, Δ26Spromoter, pBR322 ori, NP-Gn/Gc-epitope	This study
pET28(+)	IPTG-inducible, T7 expression vector, C-terminal 6× histidine tag, Kan^R^	Lab stock
***S. Typhimurium***		
JOL2500	Δ*lon* Δ*cpxR* Δ*sifA* Δ*asd* mutant of *S.* Typhimurium	Lab stock
JOL2420	JOL2500 containing pJHL204, vector control	This study
JOL2424	JOL2500 containing pJHL204-NP-Gn/Gc-epitope	This study
JOL2425	JOL2500 containing pJHL204-NP-P2A-Gn/Gc	This study
JOL2426	JOL2500 containing pJHL204-NS	This study
***E. coli***		
DH5α	F^-endA1^, glnV44, thi-1, recA1, relA1, gyrA96, deoR, nupG, Φ80d lacZ ΔM15 Δ(lacZYA-argF)U169, hsdR17(rK- mK^+^), λ^–^	Lab stock
BL21 (DE3)	F^–^, ompT, hsdS_B_ (r_B_^−^, m_B_^–^), dcm, gal, λ (DE3)	Lab stock
*E.coli* 232	F^–^, k^–^ u80 D(lacZYA-argF) endA1 recA1 hadR17 deoR thi-1 glnV44 gyrA96 relA1 DasdA4	Lab stock
**Primer**		
INF-γ-FW	TCAAGTGGCATAGATGTGGAAGAA	[44]
INF-γ-RV	TGGCTCTGCAGGATTTTCATG	
TNF-α-FW	CATCTTCTCAAAATTCGAGTGACAA	[44]
TNF-α-RV	TGGGAGTAGACAAGGTACAACCC	
IL-4-FW	ACAGGAGAAGGGACGCCAT	[44]
IL-4-RV	GAAGCCCTACAGACGAGCTCA	
IL-6-FW	CAGAATTGCCATCGTACAACTCTTTTCTCA	[44]
IL-6-RV	AAGTGCATCATCGTTGTTCATACA	
IL-10-FW	GGTTGCCAAGCCTTATCGGA	[44]
IL-10-RV	ACCTGCTCCACTGCCTTGCT	
β-actin-FW	AGAGGGAAATCGTGCGTGAC	[44]
β-actin-RV	CAATAGTGATGACCTGGCCGT	
S-segment-FW	GGGTCCCTGAAGGAGTTGTAAA	[46]
S-segment-RV	TGCCTTCACCAAGACTATCAATGT	

FW—forward primer; RW—reverse primer.

**Table 2:** Experiment schedule for immunization.

Group (*n* = 8)	Bacterial Strain	Route	Dosage (CFU/100 µL)	Booster	Serum Collection
A	PBS	i.m	1 × 10^0^	1 × 10^0^	0, 1, 2, 4, 6, 8 weeks post immunization
B	JOL2420	i.m	1 × 10^7^	1 × 10^7^	
C	JOL2424	i.m	1 × 10^7^	1 × 10^7^	
D	JOL2425	i.m	1 × 10^7^	1 × 10^7^	
E	JOL2426	i.m	1 × 10^7^	1 × 10^7^	

i.m: intra muscular route.

### 2.10. MTT Assay

The proliferative response following immunization was assessed by 3-(4, 5-dimethylthiazol-2-yl)-2, 5-diphenyl tetrazolium bromide (MTT, Sigma-Aldrich) assay. Isolated splenocytes were seeded in 96-well plates (1 × 10^5^ cells/well) and stimulated with purified NP-Gn/Gc-epitope, NP, Gn/Gc, and NS recombinant proteins for 48 h, respectively. Next, cells were treated with MTT reagent (5 mg/mL) and incubated for 1 h at 37 °C. The MTT crystals were solubilized with 100 μL of DMSO (Sigma-Aldrich), and the plates were placed on a shaker to solubilize the formation of purple crystal formazan. Absorption was measured using a microplate reader at a wavelength of 590 nm. 

### 2.11. Fluorescence-Activated Cell Sorting Analysis 

Following immunization, the magnitude of the splenic T cell differentiation was assessed by fluorescence-activated cell sorting assay (FACS). Briefly, 1 × 10^5^ viable cells were seeded in 96-well plates, stimulated with purified NP-Gn/Gc-epitope, NP, Gn/Gc, and NS recombinant proteins for 48 h, and incubated at 37 °C. After 72 h, cells were stained with anti-mouse CD3a-PE (Miltenyi Biotec, Bergisch-Gladbach, Germany), CD4-perCPvio700 (Miltenyi Biotec), and CD8a-FITC (Miltenyi Biotec) antibodies (8 µg/mL) for 30 min at 4 °C in the dark. Finally, cells were washed with FACS running buffer (Miltenyi Biotec), and the T-cell populations CD3^+^CD4^+^ and CD3^+^CD8^+^ were gated from the CD3^+^ population and analyzed using the MacsQuant analysis system (Miltenyi Biotec).

### 2.12. Enzyme-Linked Immunosorbent Assay 

Antigen-specific immunoglobulin (Ig) G and IgM levels were measured by indirect ELISA using serum samples collected on weeks 0, 1, 2, 4, 6, and 8 post-primary vaccination. Briefly, purified antigens were coated (500 ng/well) on 96-well high-binding polystyrene plates (Greiner Bio-One, Kremsmuster, Austria) and incubated at 4 °C overnight. The following day, wells were blocked with 5% skim milk (BD Difco) for 1 h at room temperature and washed three times with 0.1% phosphate-buffered saline with tween 20 (PBSt). Next, wells were incubated with serum samples (1:50) for 1 h at 37 °C, and plates were washed three times with PBSt. HRP-conjugated goat anti-mouse IgG and IgM (Southern biotech, AL, USA) secondary antibodies at 1:3000 dilution were added, and the plates were incubated at 37 °C for 1 h. After washing three times with PBSt, Color development was achieved with the prepared O-phenylenediamine dihydrochloride (OPD, Sigma-Aldrich) substrate in phosphate-citrate buffer containing H_2_O_2_ at room temperature in the dark. Optical density (OD) was measured at 492 nm using an automated ELISA spectrophotometer (Tecan, Groedig, Austria).

### 2.13. Focus Reduction Neutralization Test 

To determine the titers of neutralizing antibodies against SFTSV, a focus reduction neutralization test (FRNT_50_) assay was performed. Mice sera were collected at week 2 post-booster immunization, inactivated at 56 °C for 30 min, and diluted in 2-fold increments. Each dilution was mixed with an equal volume of 200 FAID_50_ SFTSV and incubated for 1 h at 37 °C. The mixture was inoculated into Vero E6 cells in 96-well plates and incubated at 37 °C for 1 h. After infection, cells were overlaid with DMEM containing 4% FBS, and the plates were incubated at 37 °C in 5% CO_2_. After 5 days post-incubation, cells were fixed with 80% cold acetone and incubated with anti-NP polyclonal antibodies, followed by incubation with anti-rabbit-alexa 488-IgG secondary antibody (Invitrogen). Fluorescence foci were observed using the Leica Fluorescence microscope (Leica Biosystems, Nussloch, Germany). The antibody titers were expressed as reciprocals of the highest serum dilution, showing a 50% fluorescence reduction.

### 2.14. Quantification of Viral Copy Numbers by qRT-PCR

Following the SFTSV challenge, total RNA was isolated from the serum and homogenized organ samples using the AccuPrep Viral RNA Extraction Kit (Bioneer, Daejeon, Republic of Korea) and GeneAll Hybrid-R kit according to the manufacturer’s instructions, respectively. Viral copy numbers were examined by quantitative RT-PCR. SFTSV-specific primers were designed based on the S segment [46], and the copy numbers were calculated as a ratio based on the standard control [46].

### 2.15. Histopathology

After the mouse challenge, four mice from each group were sacrificed at 4 days post-infection (dpi); spleen and liver samples were collected from these mice. The tissues were fixed with 10% formalin, embedded with paraffin, and sectioned to 3 µm thickness using a microtome (Thermo Fisher Scientific). The sectioned tissues were stained with hematoxylin and eosin (H&E) according to a standard protocol and examined by light microscopy (Axio Imager2, Zeiss, Oberkochen, Germany).

### 2.16. Statistical Analysis

All data analyses were performed with Prism software 8.0 (GraphPad Software, Boston, MA, USA). Values were expressed as the means ± standard deviations (SDs). Statistical analyses were carried out using Student’s t-test and ANOVA. The asterisks indicate significant differences among the groups (* *p* ≤ 0.05, ** *p* ≤ 0.01, *** *p* ≤ 0.005, and **** *p* ≤ 0.0001).

## 3. Results

### 3.1. Validation and Construction of the Recombinant Eukaryotic Expression Vector Expressing the SFTSV Antigenic Genes 

**Figure 1:**
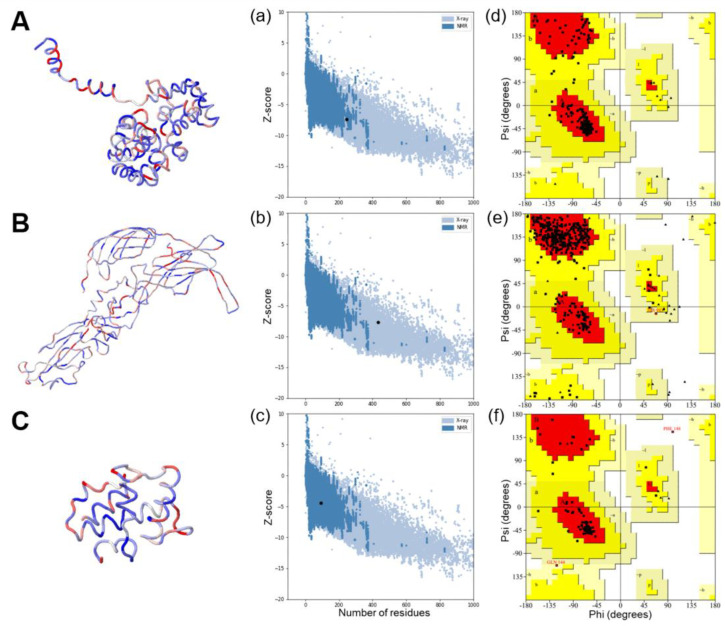
Characterization of vaccine antigens. (**A**–**C**) Predicted 3D structure of vaccine constructs. (**a**–**c**) ProSA web with z-score indicates overall model quality and recognizes errors in 3D model of vaccine antigens. (**d**–**f**) Ramachandran plot describes the validation of the vaccine construct by showing amino acids in favored and allowed regions.

**Figure 2:**
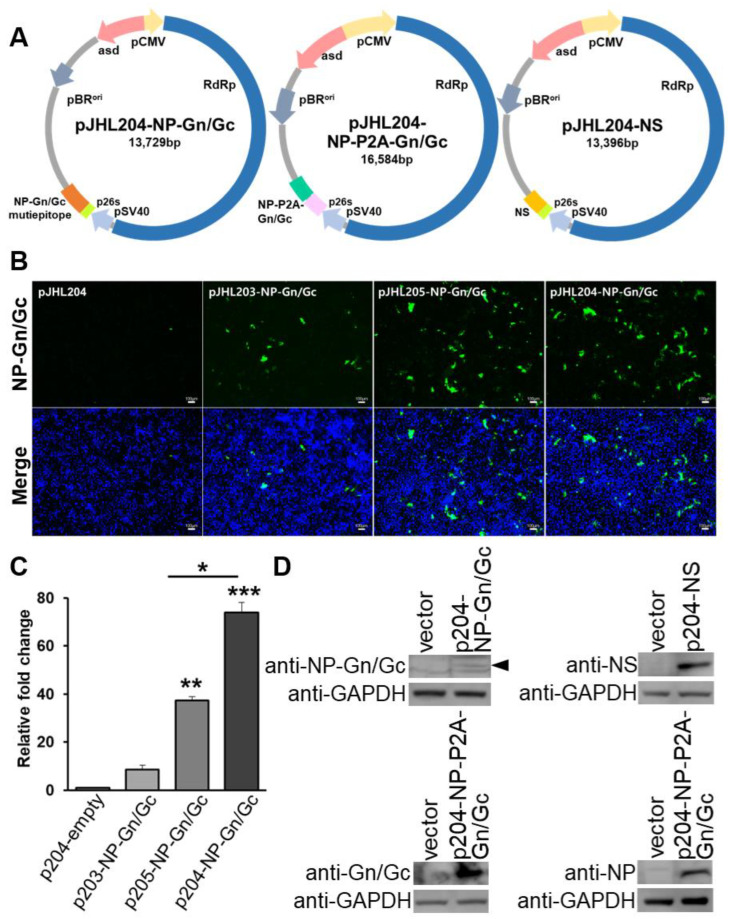
Construction of the recombinant eukaryotic expression vector. (**A**) Graphical representation of the recombinant SFTSV vaccine constructs. (**B**) HEK293T was transfected with expression plasmids: pJHL204-empty, pJHL203-NP-GnGc-epitope, pJHL205-NP-GnGc-epitope, pJHL204-NP, and GnGc-epitope. Cells were fixed and incubated with anti-NP-GnGc-epitope antibodies and labeled with alexafluor-488-labeled anti-rabbit IgG (green); nuclei were counterstained with DAPI (blue). Scale bars, 100 μm. (**C**) Relative mRNA expression of transfected HEK293T cells was evaluated by qRT-PCR using NP-specific primer, and relative gene expression was analyzed by the 2^−ΔΔCT^ method. *p*-values indicate statistical significance (* *p* ≤ 0.05, ** *p* ≤ 0.001, and *** *p* ≤ 0.005). (**D**) HEK293T cells were transfected with indicated plasmids, and the cell lysate was subjected to Western blot analysis. Protein bands were detected at 48 kDa (arrow), 28 kDa, 50 kDa, and 30 kDa, corresponding to the expression of NP-GnGc-epitope, NP, Gn/Gc, and NS, respectively.

Recombinant SFTS vaccine constructs encoding NP, NSs, and Gn/Gc proteins were selected based on the consensus sequence of 12 clinical isolates from patients infected with SFTSV. The synthesized genes were composed of full-length NP and Gn/Gc epitope fused with a GSAGSA linker, full-length NP linked to Gn/Gc with a P2A peptide, and full-length NSs. The tertiary structure of the selected antigens was validated using ProSA and Procheck web servers. The ProSA server analysis revealed that NP-Gn/Gc-epitope, NP-P2A-Gn/Gc, and NS as vaccine antigens had z-scores of −7.43, −7.71, and −4.47, respectively (Figure 1a–c). The Ramachandran plot analysis demonstrated that most of the amino acids of selected vaccine antigens were plotted in the core and favored regions (Figure 1d–f). The selected and validated antigens were cloned into the pJHL204 eukaryotic expression vector system (Figure 2A). The plasmids harboring three SFTSV antigens were then transformed into an attenuated *Salmonella* mutant strain, JOL 2500, and confirmed by PCR (Supplemental Figure S2). The role of SV40 and 26SP in antigen expression was evaluated by IFA. Our data indicated that cells transfected with pJHL204 plasmid with intact SV40 and 26SP showed an enhanced green fluorescence signal and significantly increased NP-specific mRNA level (>60 times) (Figure 2B,C), confirming the direct role of SV40 and 26SP in antigen expression. 

### 3.2. SFTSV Antigen Expression Confirmation 

We tested the expression of NP-Gn/Gc epitope, NP, Gn/Gc, and NS antigens from each plasmid by Western blot assay using specific antibodies directed against each antigen. This analysis showed reactive bands at 48 kDa, 28k Da, 50 kDa, and 30 kDa corresponding to the NP-Gn/Gc epitope, NP, Gn/Gc, and NS antigens, respectively (Figure 2D). 

### 3.3. Cell-Mediated Immune Responses in Mice Immunized with SFTSV Vaccine Constructs 

**Figure 3:**
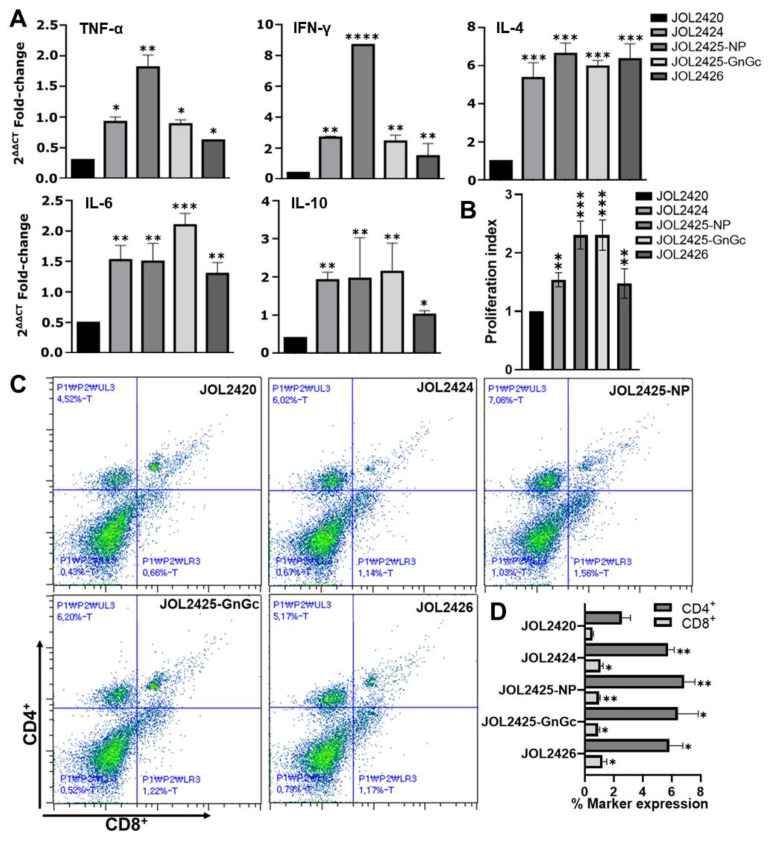
Constructs for the SFTSV vaccine elicit cellular immune reactions. Two-week post-booster immunization splenocytes were collected and stimulated with each antigen. (**A**) At 48 h post-stimulation, changes in Th1 and Th2 cytokine profiles were assessed by qRT-PCR. (**B**) Splenocyte proliferation indices in immunized mice were compared with the control group. (**C**) Representative scatter plot diagrams of CD4^+^ and CD8^+^ sub-T cell populations were gated from a CD3^+^ population. (**D**) Upon stimulation with SFTSV antigens, the percentages in CD4^+^ and CD8^+^ T cell subpopulations were evaluated by flow cytometry. * *p* ≤ 0.05, ** *p* ≤ 0.001, *** *p* ≤ 0.005, and **** *p* ≤ 0.0001 indicate significant differences compared to the vector control. Error bar: mean ± S.D.

An effective vaccine should induce high cellular immune responses to eliminate intracellular pathogens. Our results indicated that mice in all immunized groups induced significantly higher Th1 cytokines IL-4 and IL-10, IFN-γ and Th2 cytokines IL-6, and TNF-α and secretion than the vector control group. However, mice immunized with JOL2425 presented higher NP and Gn/GC-specific IL-4, TNF-α, IFN-γ, and IL-6 transcription levels (Figure 3A). These results suggest that constructed strains induced mixed Th1/Th2-based immune responses.

Furthermore, we evaluated the proliferation index of T cells following immunization. The results revealed that mice immunized with vaccine strains induced significantly higher T cell proliferation than those immunized with the vector control. Mice immunized with JOL2425 also generated significantly higher NP- and Gn/Gc-specific proliferation indices (Figure 3B). 

To characterize the T cell differentiation in immunized mice, we analyzed CD4+ and CD8+ T cell populations by flow cytometry (Figure 3C,D). We observed a substantial increase in CD4^+^ and CD8^+^ T cell subpopulations in mice immunized with JOL2425 compared with vector control. Furthermore, mice immunized with JOL2426 showed a significant increase in the CD8^+^ T cell population (Figure 3D). 

### 3.4. The Constructs Induced Robust Humoral Immune Response and Neutralizing Antibodies against SFTSV 

**Figure 4:**
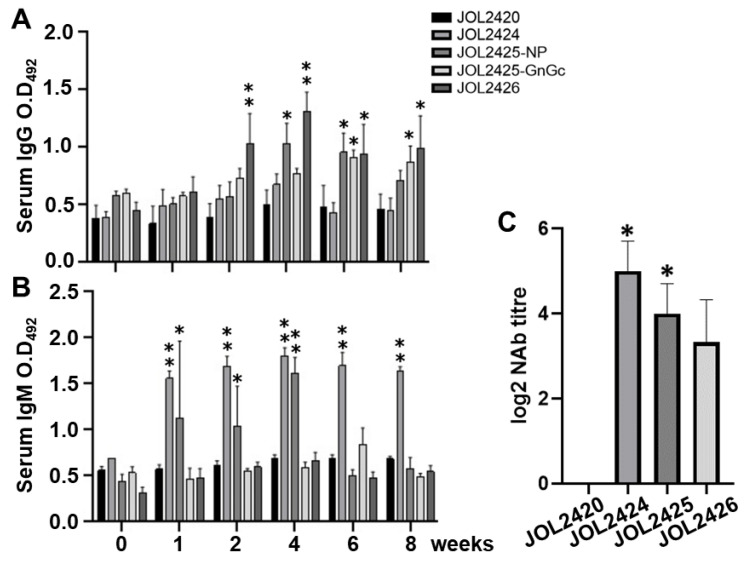
SFTSV vaccine constructs induce humoral immune responses and potent neutralizing antibodies. (**A**,**B**) Antigen-specific IgG and IgM antibody responses were assessed in serum samples collected at 0, 1, 2, 4, 6, and 8 week(s) post-immunization. * *p* ≤ 0.05 and ** *p* ≤ 0.01 indicate significant differences in comparison to vector control. (**C**) Neutralizing antibody titers generated by vaccine constructs against SFTSV were determined by FRNT_50_. Individual serum samples were diluted 2-fold. Data were analyzed by unpaired Student’s t-test, and * *p* ≤ 0.05 indicates significant difference in comparison to vector control.

The expression of Th2 cytokines IL-6 and TNF-α in immunized mice indicates the induction of the humoral response (Figure 3A). As a direct and accurate determination of humoral immune responses, antigen-specific IgG and IgM levels in immunized serum samples were examined by indirect ELISA. After the 2-week post-booster immunization, mice immunized with JOL2425 and JOL2426 induced significantly higher IgG antibody levels than the control group (Figure 4A); mice immunized with JOL2424 showed substantially higher serum IgM levels (Figure 4B). These results correlate with the induction of neutralizing antibodies in which immunized mice generated higher neutralizing titers against SFTSV. Notably, mice immunized with JOL2424 and JOL2425 had neutralization titers of 5.0 and 4.0 FRNT_50_ (log_2_), respectively (Figure 4C). Our results suggest that mice immunized with JOL2424 and JOL2425 effectively induced both humoral and neutralizing antibody responses against SFTSV.

### 3.5. Evaluation of Protective Efficacy of Constructs against SFTSV in an AAV-DC-SIGN-Transduced Mouse Model

**Figure 5:**
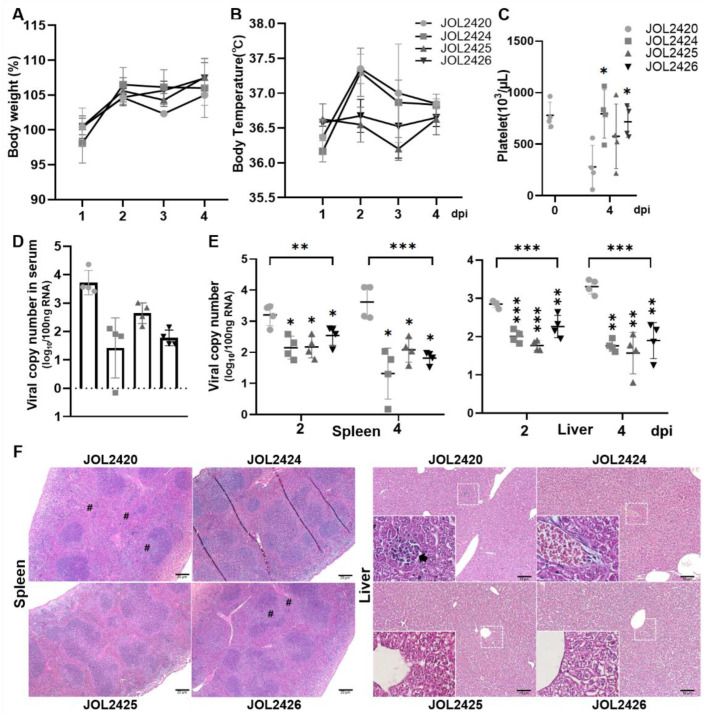
The protective effects of the vaccines were assessed in an hDC-SIGN transduced murine model. AAV-hDC-SIGN-transduced mice were examined daily during the four-day time course following challenge with 1 × 10^3^ FAID50 of SFTSV (*n* = 4 per group). (**A**) Body weights and (**B**) temperature were measured every day, and (**C**) blood cell counts were conducted using automated blood cell counter and analyzed the platelet count. (**D**) Viral RNA copy number was determined by qRT-PCR from serum at 4 dpi, (**E**) spleen and liver collected at 2 and 4 dpi. One-way ANOVA and unpaired Student’s *t*-test were used to determine the level of statistical significance. Significant differences were compared between vaccine and vector control groups. * *p* ≤ 0.05, ** *p* ≤ 0.001, *** *p* ≤ 0.005, (**F**) Representative sections (*n* = 4 per group) of challenged spleen and liver tissue were analyzed by H&E staining. Images of the spleen (magnification: 5×) and liver (magnification: 10× and 40×) were taken under the microscope. Decreased white pulp area (#) and infiltration of mononuclear cells (arrow) were observed in spleen and liver samples, respectively. Scale bars, 20 μm (**left panel**) and 10 μm (**right panel**).

We investigated the protective efficacy of the constructs using an hDC-SIGN-transduced mouse model. One week after transduction, each group of mice (*n* = 8) were challenged with 1 × 10^3^ FAID_50_ SFTSV and sacrificed on days 2 (*n* = 4) or 4 (*n* = 4) post-infection. Mice immunized with JOL2420 and JOL2424 showed slightly increased body temperature. In contrast, mice immunized with JOL2425 and JOL2426 did not present noticeable variations in body temperature during the experiment (Figure 5A). In addition, we did not observe a significant reduction in body weight among mice in any of the immunized groups. However, mice in the PBS group experienced a considerable weight loss from 1 to 3 dpi (Figure 5B). All immunized mice (JOL2424, JOL2425, and JOL2426) maintained average platelet counts following the SFTSV challenge, but a significantly reduced platelet (PLT) count was seen at 4 dpi in the control group (JOL2420). The expected reference range of PLT in normal laboratory mice is 668–1543 × 10^3^/μL [47] (Figure 5C). In addition, viral load in serum, spleen, and liver samples were determined by qRT-PCR. Compared to the control group, viral copy numbers in the serum (Figure 5D), spleen, and liver (Figure 5E) samples were significantly reduced in all immunized groups (*p* ≤ 0.001 and *p* ≤ 0.005). Furthermore, mice in the all-immunized group showed reduced liver necrosis or hepatocyte degeneration and reduced loss of white pulp in the spleen (Figure 5F).

## 4. Discussion and Conclusions

Several vaccine candidates exploiting various strategies against SFTS have been studied, including live attenuated vaccines [48], DNA vaccines [2], and viral vector vaccines [49,50]. However, these strategies have drawbacks, including weak immunogenicity, lack of target specificity, and non-active delivery of plasmids [51]. Over the past few decades, live-attenuated *Salmonella* has been used to deliver homologous and heterologous antigens against various pathogens. *Salmonella* entering the host system is recognized by its pathogen-related molecular patterns (PAMP) and triggers host innate and adaptive immune responses; hence, it does not require any adjuvants or formulations. In this study, we utilized an attenuated *Salmonella* strain, JOL2500 (Δ*lon*, Δ*cpxR*, Δ*sifA*, Δ*asd*), to deliver a eukaryotic vector pJHL204 expressing multiple SFTSV antigens, NP, Gn/Gc, and NS to combat emerging SFTS. 

The Gn and Gc, the two main antigenic glycoproteins of SFTSV, are considered key targets for neutralizing antibodies. When designing our vaccine constructs, we selected an immunodominant epitope of the Gn/Gc glycoprotein and connected it to the Np protein with a linker to be expressed as a multi-cistronic antigen [11,52]. In a different vaccine construct, we introduced a P2A peptide between full-length NP and Gn/Gc genes to mediate multimetric antigens expression and post-translational separation of antigens via the self-cleaving activity of P2A [53,54]. The structural stability of selected and designed vaccine antigens was confirmed by analyzing the stereochemical properties using the Ramachandran plot analysis (Figure 1). The antigens were cloned into the pJHL204 vector in-frame with the RdRp complex (nsp1–4). The expression of each antigen delivered by attenuated *Salmonella* strain, JOL2500, was confirmed by Western blot (Figure 2A,D). Including the RdRp complex in the pJHL204 vector will increase the antigen expression by identifying the 26SP region located upstream of the MCS, which will lead to robust amplification of cytoplasmic mRNA [29].

Cytokine profiling confirmed that mice immunized with strains JOL2424 (NP-Gn/Gc-epitope), JOL2425 (NP-P2A-Gn/Gc), and JOL2426 (NS) induced Th1/Th2-based immune responses (Figure 3A). The upregulated IFN-γ plays a significant role in curbing host immune response by promoting major histocompatibility complex (MHC)-I antigen presentation and activation of T-helper cell responses [55]. We also observed that NP protein upregulated the cytokine IL-4 secretion in mice immunized with JOL2425, indicating stimulation of Th2 cells which will activate B cells to produce IgG [56]. This balanced Th1/Th2-based immunity induced by vaccine constructs will play a pivotal role in fighting against viral infectious diseases. The T-cell responses are considered the central immune mechanism against intracellular pathogens [57,58]. Mice immunized with JOL2424, JOL2425, and JOL2426 induced strong antigen-specific CD4^+^ T and CD8^+^ T cell response. However, we observed comparatively higher NS-specific CD8^+^ T cell response in mice immunized with JOL2426 (Figure 3C). This result is correlated with a previous study, where a higher NS-specific CD8+ T cell response was observed in mice immunized with a DNA vaccine expressing NP and NS [59]. The dramatic loss of T cells in SFTSV-infected patients directly affects viral clearance due to inadequate phagocytosis of virus-infected cells [60]. Therefore, the vaccine constructs exhibited a cell-mediated response that can stimulate CD4^+^ and CD8^+^ T cells to promote antigen-specific neutralizing antibody production and viral clearance by activating cytotoxic T cells, respectively [61,62]. 

In the host cells, attenuated *Salmonella*-delivering antigens will be phagocytosed by antigen-presenting cells, primarily macrophages, stimulating antigen-specific B and T cell responses [63,64]. Mice immunized with vaccine constructs JOL2425 and 2426 induced a strong IgG response, whereas JOL2424 and JOL2425 generated a strong IgM response (Figure 4). Interestingly, we observe anti-NP specific IgM response in mice immunized with JOL2424 and JOL2425 (Figure 4B). The IgM antibodies produced by the highly immunogenic NP protein of SFTSV play a significant role in viral clearance, mainly during early infection before the IgG responses occur. Subsequently, memory IgG antibodies with increased affinity are produced after maturation and isotype class-switching [65]. This indicates that the coordinated humoral response to both anti-NP IgM and IgG antibodies is associated with increased protective immunity. Vaccines based on Gn/Gc and NS antigens produce a relatively higher IgG immune response but not adequate IgM, which was evident in mice immunized with JOL2425 and JOL2426, respectively (Figure 4B). Antigen-specific antibodies produced by B lymphocytes arrest viral replication by blocking the viral attachment to the host cells [66]. Mice immunized with JOL2424 and JOL2425 induce a significantly higher neutralization titer (Figure 4C). A similar neutralizing antibody titer was observed in mice immunized with an inactivated SFTSV vaccine suggesting the efficacy of our designed vaccine constructs [8,32,67,68]. The higher virus-neutralizing activity was related to this coordinated IgM and IgG response against NP and GnGc, which may contribute to protective immunity. These results suggest that our designed constructs via attenuated *Salmonella* were able to produce strong IgG and IgM antibodies and neutralizing antibodies to emphasize the possibility of a vaccination strategy against SFTSV. 

The receptor usage of bunyaviruses is not fully understood. The lectin dendritic-cell-specific intercellular adhesion molecule 3-grabbing nonintegrin (DC-SIGN) can serve as a receptor for phleboviruses to facilitate viral entry [35]. One study reported that Gn/Gc protein SFTSV utilizes DC-SIGN as the receptor for host entry [34]. To overcome the unavailability of a humanized mouse model for SFTSV infection, we developed a transgenic mouse model by transducing mice with an adeno-associated vector expressing the human DC-SIGN gene. We observed that overexpression of hDC-SIGN facilitated SFTSV entry and replication in several cell lines. Moreover, we demonstrated that administration of rAAV-hDC-SIGN resulted in robust hDC-SIGN expression in multiple organs of mice and permitted successful replication of a clinical SFTSV isolate [30]. Following the challenge, mice immunized with JOL2425 and JOL2426 showed significantly reduced viral loads in the spleen and liver. In addition, we did not observe any significant fluctuations in platelet count, body weight, and temperature in immunized mice (Figure 5). 

In summary, we demonstrated that the *Salmonella*-mediated SFTS vaccine constructs efficiently induced humoral and cell-mediated immune responses in C57BL/6 mice. For this demonstration, we used a eukaryotic self-mRNA replicating vector, pJHL204, expressing multiple SFTS viral antigenic genes for NP and Gn/Gc. In addition, these constructs could generate strong neutralizing antibodies against SFTSV and protect the transduced mice against SFTSV infection. In particular, the viral load in serum and organ samples was significantly reduced in the immunized mice. This suggests that this *Salmonella* system expressing multiple SFTSV antigens is a new avenue for developing a vaccine intervention to combat the SFTS virus.

## Data Availability

Data are available from the authors upon reasonable request.

## Supplementary Materials

The following supporting information can be downloaded at https://www.mdpi.com/article/10.3390/pharmaceutics15051339/s1. Table S1: Experiment Schedule for SFTSV challenge; Table S2: Post-challenge body temperature measurements (°C); Figure S1: The BepiPred program automatically determined the cut-off value for linear B-cell epitopes of the Gn-Gc protein; Figure S2: Vaccine antigens expressed by *Salmonella* mutant, JOL2500 was confirmed by PCR using antigen-specific primers. Genes were amplified at 2.1 kilobase pairs (kbp), 4.1 kbp, and 1.6 kbp for NP-Gn/Gc-epitope, NP-P2A-Gn/Gc, NSs genes, respectively.
