# Supplementary material for: An mRNA-Based Multiple Antigenic Gene Expression System Delivered by Engineered Salmonella for Severe Fever with Thrombocytopenia Syndrome and Assessment of Its Immunogenicity and Protection Using a Human DC-SIGN-Transduced Mouse Model
Source: Pharmaceutics. 2023 Apr 26;15(5):1339. doi: 10.3390/pharmaceutics15051339 (PMC10222543; doi:10.3390/pharmaceutics15051339)
Supplement: Supplementary file 1 [file pharmaceutics-15-01339-s001.zip › pharmaceutics-2310117-supplementary.pdf]

Supporting Information

Ji-Young Park[1], Chamith Hewawaduge[1], Khristine Kaith S. Lloren[1], Byungkwan Oh[2], Mi Young So[1], John Hwa Lee[1,*]

[1] Department of Veterinary public health, College of Veterinary Medicine, Jeonbuk National University, Iksan, 54596, Republic of Korea

[2] Department of Veterinary Pathology, College of Veterinary Medicine, Jeonbuk National University, Iksan, 54596, Republic of Korea

*Corresponding author : John Hwa Lee, DVM, Ph.D,

E-mail:johnhlee@jbnu.ac.kr. Phone : +82-63-850-0940

Supplementary table S1. Experiment Schedule for SFTSV challenge

Group (n=8)	Strain	Route	Dosage (CFU/100 µl)	Booster	AAV/hDC-SIGN transduction	Challenge
A	PBS	IM	100 ul	100 ul	1 week post booster immunization (1 x 10^{11}vp, i.v)	1×10^3 $FAID_{50}$ SFTSV (i.p)
B	JOL2420	IM	1 x 10^7	1 x 10^7		
C	JOL2424	IM	1 x 10^7	1 x 10^7		
D	JOL2425	IM	1 x 10^7	1 x 10^7		
E	JOL2426	IM	1 x 10^7	1 x 10^7		

i.v: intravenous route, i.p: intraperitoneal route

Supplementary table S2. Post-challenge body temperature measurements (°C)

	**JOL2420**	**JOL2424**	**JOL2425**	**JOL2426**
**Day 01**	36.4±0.2	36.2±0.1	36.6±0.2	36.6±0.1
**Day 02**	37.4±0.1	37.3±0.3	36.6±0.2	36.7±0.2
**Day 03**	37.0±0.5	36.9±0.3	36.2±0.1	36.5±0.4
**Day 04**	36.9±0.1	36.8±0.1	36.6±0.2	36.7±0.1

Supplemental Figure S1

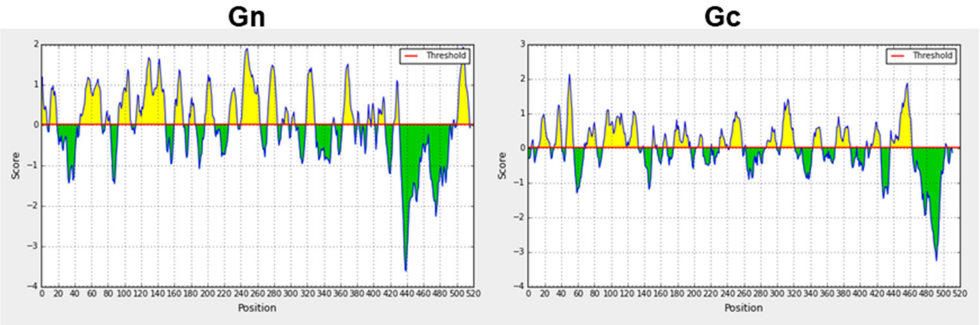

WLRNHSQFQGYVGQRGGRSQVSYYPAENSYGSAGSAGGWGCGSGK
SRTESGELCSSDGSAGSAKEGTGPCSESEEKTCKTSGSGSAGSAGA
CLHFTAPGSPDSKCLKIKVGSAGSARCTSVRRCRWAGDCQSGCPSH
FTSNSFSDDWAGKMDRAGLPTGANIPSPTDWLNALFGNG

Supplemental Figure S1. The BepiPred program automatically determined the cut-off value for linear B-cell epitopes of the Gn-Gc protein.

Supplemental Figure S2

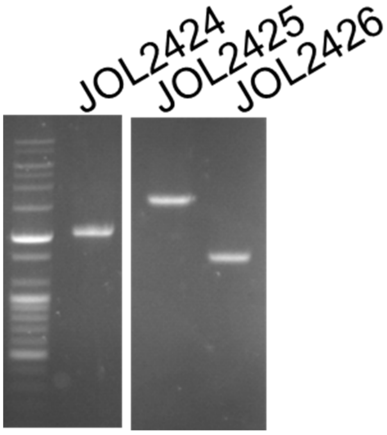

Supplemental Figure S2. Vaccine antigens expressed by *Salmonella* mutant, JOL2500 was confirmed by PCR using antigen-specific primers. Genes were amplified at 2.1 kilobase pairs (kbp), 4.1 kbp, and 1.6 kbp for NP-Gn/Gc-epitope, NP-P2A-Gn/Gc, NSs genes, respectively.
